# Supplementary material for: Targeted stabilization of Munc18‐1 function via pharmacological chaperones
Source: EMBO Mol Med. 2020 Dec 17;13(1):e12354. doi: 10.15252/emmm.202012354 (PMC7799358; doi:10.15252/emmm.202012354)
Supplement: Supplementary file 1 — Appendix [file EMMM-13-e12354-s001.pdf]

Appendix for

Targeted stabilization of Munc18-1 function via pharmacological chaperones

Debra Abramov[1], Noah Guy Lewis Guiberson[1], Andrew Daab[1,2], Yoonmi Na[1], Gregory A. Petsko[1,3], Manu Sharma[1] & Jacqueline Burré[1]

[1]Appel Institute for Alzheimer's Disease Research, Brain and Mind Research Institute, Weill Cornell Medicine, [2]current address: University of Bath, UK, [3]current address: Ann Romney Center for Neurologic Diseases, Department of Neurology, Brigham and Women's Hospital and Harvard Medical School.

Table of Contents

- Appendix Figure S1. Solubility of syntaxin-1 is reduced by mutant Munc18-1 variants.
- Appendix Figure S2. Effect of compounds on total levels of WT Munc18-1 in neurons.
- Appendix Figure S3. Purification of recombinant Munc18-1 variants.
- Appendix Figure S4. Rescue of spontaneous neurotransmitter release in cortical mouse neurons expressing Munc18-1 R406H.
- Appendix Figure S5. Distribution of pixel intensities in mouse cortical neurons expressing WT, R406H or G544D mutant Munc18-1 that were subjected to a synaptotagmin-1 antibody uptake assay.
- Appendix Figure S6. Rescue of deficits in C. elegans.
- Appendix Table S1. Summary of statistical analyses.
- Appendix Table S2. Binding site and docking scores for top compounds identified in silico that were tested for pharmacological activity.

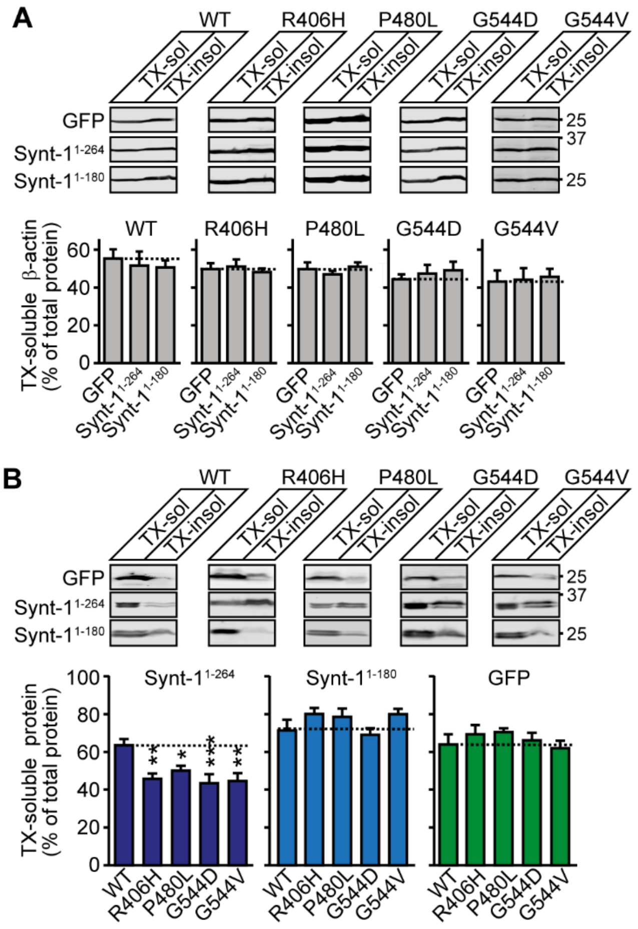

Appendix Figure S1. Solubility of syntaxin-1 is reduced by mutant Munc18-1 variants.

A Solubility of β-actin. HEK293T cells transfected with WT or mutant Munc18-1 variants and either GFP, syntaxin-$1^{1\text{-}264}$ or syntaxin-$1^{1\text{-}180}$ were solubilized in 0.1% Triton X-100 (TX), and equal volumes of soluble and insoluble fractions were analyzed by quantitative immunoblotting. TX-soluble β-actin (control) was measured as percent of total β-actin by quantitative immunoblotting. Data are means ± SEM (n = 6-7 independent experiments; exact n and p values are shown in Appendix Table S1).

B Same as in (A) except that TX-solubility of the two syntaxin-1 fragments and GFP was analyzed as a function of Munc18-1 variants. Data are means ± SEM (*$p < 0.05$, **$p < 0.01$, ***$p < 0.001$ by one-way ANOVA and Bonferroni post-hoc test; n = 6-8 independent experiments; exact n and p values are shown in Appendix Table S1).

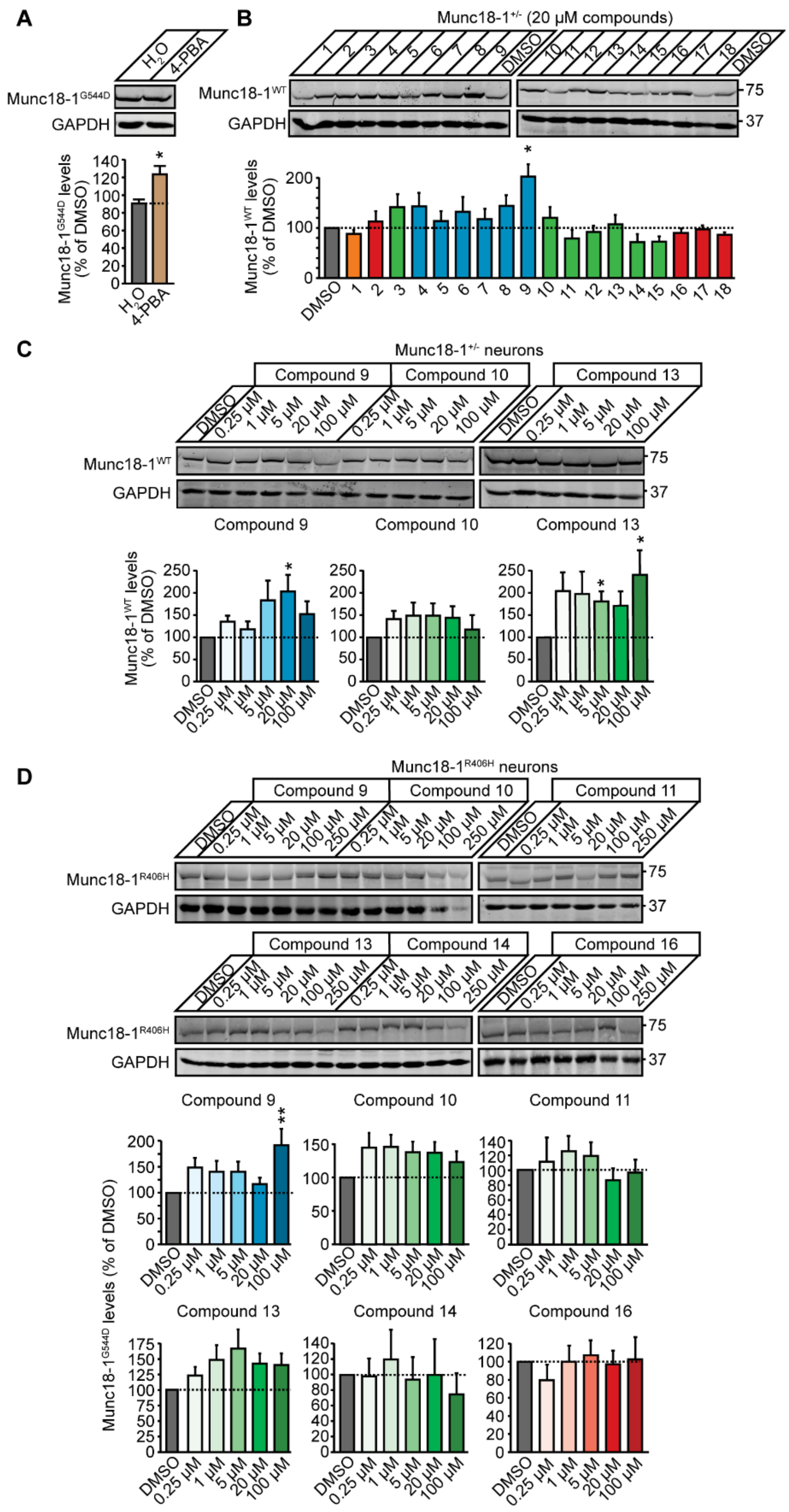

Appendix Figure S2. Effect of compounds on total levels of WT Munc18-1 in neurons.

A Total protein levels of G544D Munc18-1. G544D Munc18-1b was expressed in primary cortical mouse neurons infected with lentiviral vectors expressing cre recombinase in the

presence or absence of compounds at 4-phenylbutyrate (4-PBA), or H_2O (vehicle control). Total Munc18-1 levels were analyzed by quantitative immunoblotting 9 days after infections, normalized to GAPDH levels. Data are means ± SEM (*$p < 0.05$ by Student's t test; n = 2-5 independent experiments; exact n and p values are shown in Appendix Table S1).

B Total protein levels in hemizygous Munc18-1 neurons were analyzed by quantitative immunoblotting at 14 days in vitro, normalized to GAPDH levels. Data are means ± SEM (*$p < 0.05$ by one-way ANOVA and Bonferroni post-hoc test; n = 10 independent experiments; exact n and p values are shown in Appendix Table S1).

C Same as in B, except that DMSO (vehicle control) or compounds 9, 10 and 13 were added at 0.25, 1, 5, 20 and 100 μM. Data are means ± SEM (*$p < 0.05$ by one-way ANOVA and Dunnett post-hoc test or Kruskal-Wallis test and Dunn's multiple comparison test; n = 10-11 independent experiments; exact n and p values are shown in Appendix Table S1).

D Total protein levels of R406H Munc18-1. R406H Munc18-1b was expressed in primary cortical mouse neurons infected with lentiviral vectors expressing cre recombinase in the presence or absence of compounds 9, 10, 11, 13, 14, or 16 at indicated concentrations, or DMSO (vehicle control). Total Munc18-1 levels were analyzed by quantitative immunoblotting 9 days after infections, normalized to GAPDH levels. Data are means ± SEM (*$p < 0.05$ by Kruskal-Wallis test and Dunn's multiple comparison test; n = 7-18 independent experiments; exact n and p values are shown in Appendix Table S1).

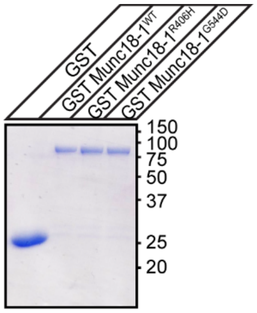

Appendix Figure S3. Purification of recombinant Munc18-1 variants.

Purification of recombinant GST, GST-tagged WT, R406H or G544D Munc18-1. SDS-PAGE analysis of 10 µg of recombinant purified protein.

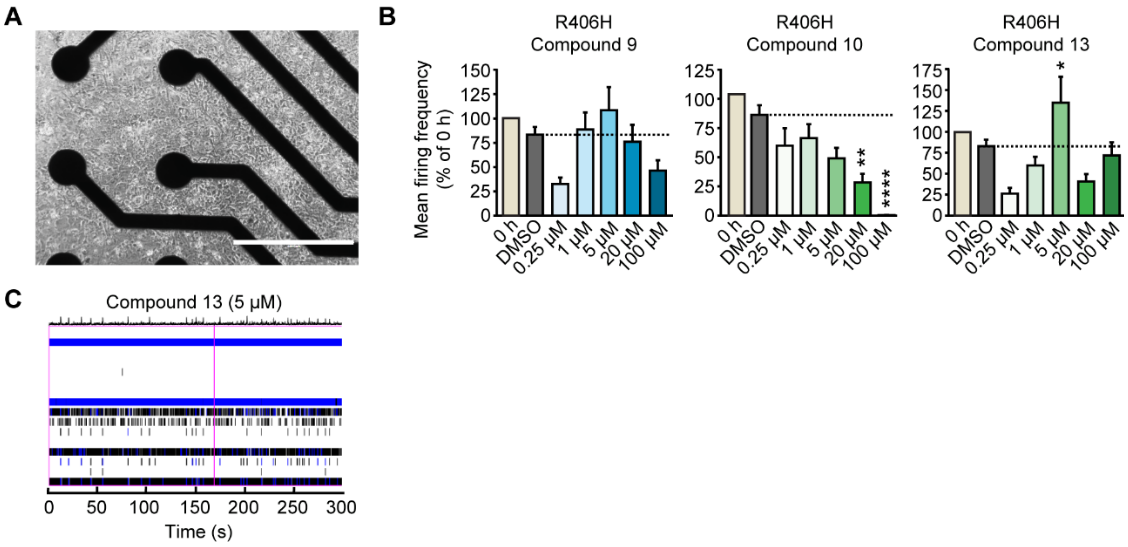

Appendix Figure S4. Rescue of spontaneous neurotransmitter release in cortical mouse neurons expressing R406H Munc18-1.

A Image of primary neurons that were plated on a multi-electrode array. Scale bar, 400 µm.

B Munc18-1 knockout neurons expressing R406H Munc18-1 were subjected to analysis of mean firing rate before addition of compounds (0 h) or 48 hours after vehicle (DMSO) or compound addition. Data are means ± SEM (*$p < 0.05$, **$p < 0.01$, ****$p < 0.0001$ by Kruskal-Wallis test and Dunn's multiple comparison, or one-way ANOVA and Dunnett's post-hoc test; n = 12-25 independent experiments; exact n and p values are shown in Appendix Table S1).

C Example of mean neuronal firing for neurons expressing R406H Munc18-1 treated with compound 13 at 5 µM.

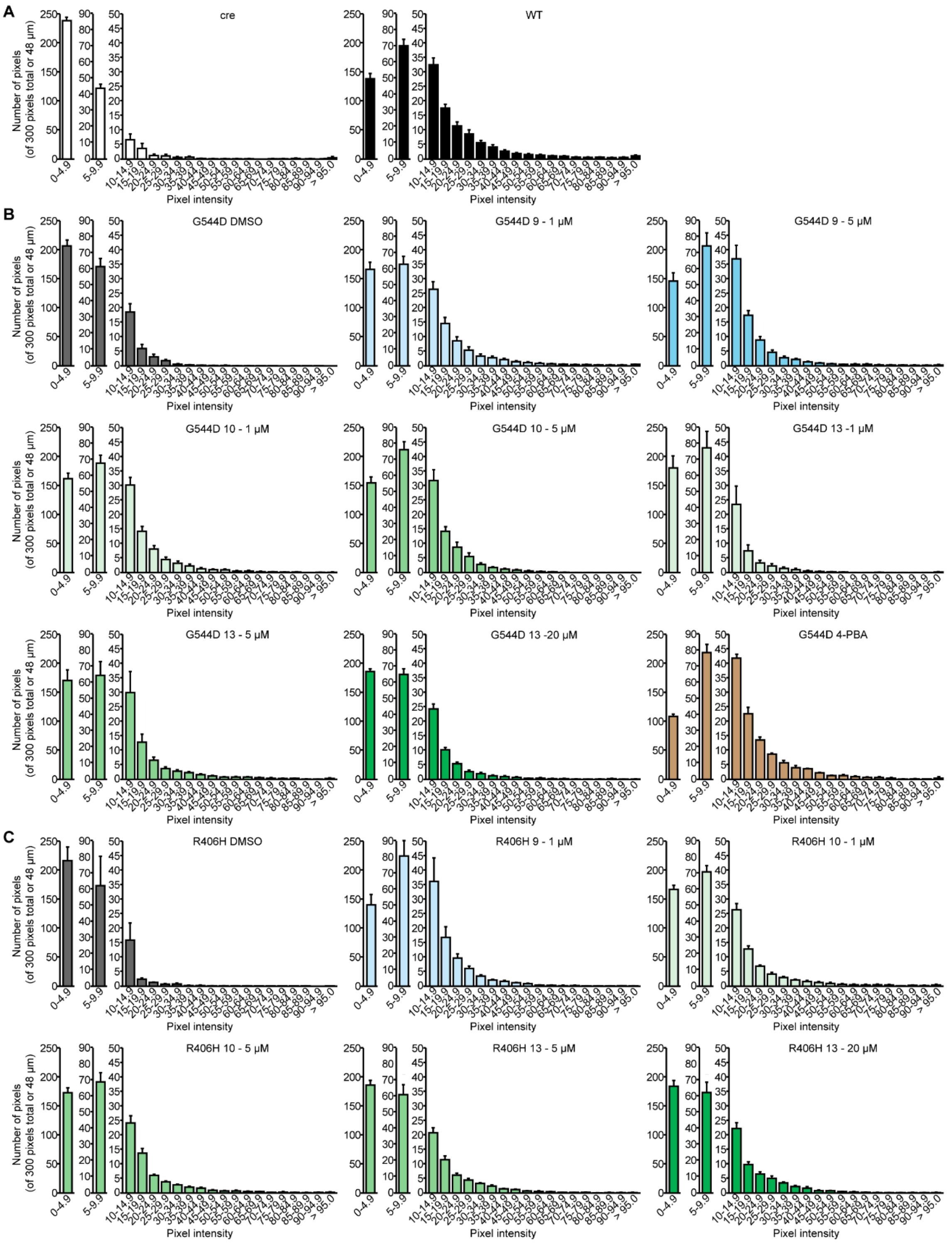

Appendix Figure S5. Distribution of pixel intensities in mouse cortical neurons expressing WT, R406H or G544D mutant Munc18-1 that were subjected to a synaptotagmin-1 antibody uptake assay.

A-C Mouse cortical neurons infected at 6 DIV with lentivirus expressing cre recombinase and/or WT (A), G544D Munc18-1b (B), or R406H Munc18-1b (C) were subjected to an

antibody uptake assay at 13 DIV after 48 h incubation with compound or vehicle control. Endocytosed synaptotagmin-1 antibody was quantified by immunostaining (Figure 6), via counting the number of pixels in pixel intensity bins of 5. Data are means ± SEM (n = 3-10 independent experiments; exact n and p values are shown in Appendix Table S1 for Figure 6).

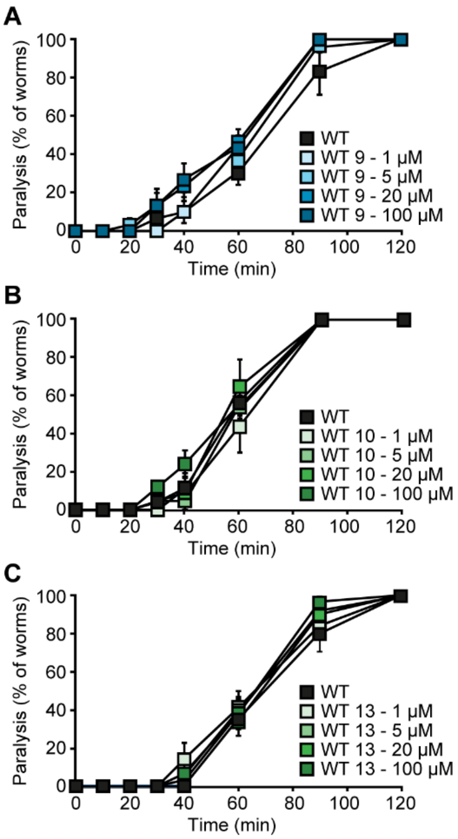

Appendix Figure S6. Rescue of deficits in *C. elegans*.

A-C Heat-induced paralysis of wild-type worms. Wild-type (WT) worms maintained in compounds at indicated concentrations were exposed to 37 °C over a period of 120 min, and paralysis was scored at indicated time points. Data are means ± SEM (n = 4 independent experiments on ten worms per experiment; exact p values are shown in Appendix Table S1).

Appendix Table S1. Summary of statistical analyses.

Figure	Dataset	Normality test	Statistical test	Multiple comparison test	n	ANOVA p value	Multi-comparison p-value
1C	1-264	failed test for normality	Kruskal Wallis	Dunn	11	0.0008	WT: 0.0810, R406H: <0.0001, P480L: 0.0227, G544D: 0.0148, G544V: 0.0067
	1-180	failed test for normality	Kruskal Wallis	Dunn	11	<0.0001	WT: >0.9999, R406H: 0.0022, P480L: 0.0001, G544D: 0.0019, G544V: 0.0013
1D	WT	failed test for normality	Kruskal Wallis		GFP, Stx1 180: 7; Stx1 264: 8	0.9351	
	R406H	failed test for normality	Kruskal Wallis	Dunn	GFP, Stx1 264: 9; Stx1 180: 8	0.0004	1-264: 0.0008,<br>1-180: 0.0021
	P480L	normal	One-way ANOVA	Bonferroni	5	0.0009	1-264: 0.0039,<br>1-180: 0.0008
	G544D	failed test for normality	Kruskal Wallis	Dunn	GFP, Stx1 264: 7; Stx1 180: 6	0.0029	1-264: 0.02,<br>1-180: 0.0094
	G544V	normal	One-way ANOVA	Bonferroni	GFP, Stx1 264: 6; Stx1 180:5	<0.0001	1-264: <0.0001,<br>1-180: 0.0001
S1A	WT	normal	One-way ANOVA		7	0.8177	
	R406H	normal	One-way ANOVA		6	0.7951	
	P480L	normal	One-way ANOVA		6	0.5438	
	G544D	normal	One-way ANOVA		6	0.709	
	G544V	failed test for normality	Kruskal Wallis		6	0.9213	
S1B	Stx1-264	normal	One-way ANOVA	Bonferroni	8	<0.0001	R406H: 0.0030, P480L: 0.0377, G544D: 0.0007, G544V: 0.0015
	Stx1-180	normal	One-way ANOVA		8	0.2489	
	GFP	normal	One-way ANOVA		6	0.7206	
3B	Munc18-1 G544D	normal	One-way ANOVA	Bonferroni	6	0.0284	1: >0.9999, 2: >0.9999, 3: 0.0005, 4: >0.9999, 5: 0.7445, 6: >0.9999, 7: >0.9999, 8: >0.9999, 9: >0.9999, 10: >0.9999, 11: >0.9999, 12: >0.9999, 13: 0.0368, 14: >0.9999, 15: >0.9999, 16: >0.9999, 17: 0.4965, 18: >0.9999
3D	Compound 9	failed test for normality	Kruskal Wallis	Dunn	15	0.0558	0.25 µM: 0.1127, 1 µM: 0.0163, 5 µM: 0.0431, 20 µM: 0.3996, 100 µM: 0.1811
	Compound 10	normal	One-way ANOVA	Dunnett	15	0.0067	0.25 µM: 0.0052, 1 µM: 0.8067, 5 µM: 0.1896, 20 µM: 0.7528, 100 µM: 0.9997
	Compound 13	failed test for normality	Kruskal Wallis	Dunn	12	0.0036	0.25 µM: 0.0283, 1 µM: 0.0008, 5 µM: 0.1304, 20 µM: 0.0061, 100 µM: 0.63
	Compound 11	normal	One-way ANOVA		7	0.6555	
	Compound 14	normal	One-way ANOVA		7	0.6863	
	Compound 16	normal	One-way ANOVA		6	0.9532	
S2A	4-phenylbutyrate		Unpaired Student's T-test		5 for vehicle, 2 for treatment	0.0285	
S2B	Heterozygous neurons	normal	One-way ANOVA	Bonferroni	10	<0.0001	1: >0.9999, 2: >0.9999, 3: >0.9999, 4: >0.9999, 5: >0.9999, 6: >0.9999, 7: >0.9999, 8: 0.8345, 9: 0.0364, 10: >0.9999, 11: >0.9999, 12: >0.9999, 13: 0.3527, 14: >0.9999, 15: 0.3752, 16: >0.9999, 17: >0.9999, 18: 0.3962
S2C	Compound 9	normal	One-way ANOVA	Dunnett	11	0.093	0.25 µM: 0.8686, 1 µM: 0.9911, 5 µM: 0.152, 20 µM: 0.0395, 100 µM: 0.5583
	Compound 10	normal	One-way ANOVA		10	0.6267	
	Compound 13	failed test for normality	Kruskal Wallis	Dunn	10	0.0622	0.25 µM: 0.0877, 1 µM: 0.1465, 5 µM: 0.0272, 20 µM: 0.1514, 100 µM: 0.0424
S2D	Compound 9	normal	One-way ANOVA	Dunnett	18	0.0361	0.25 µM: 0.2835, 1 µM: 0.4441, 5 µM: 0.4668, 20 µM: 0.9664, 100 µM: 0.006
	Compound 10	normal	One-way ANOVA		17	0.2998	
	Compound 13	normal	One-way ANOVA		17	0.2331	
	Compound 11	normal	One-way ANOVA		7	0.4183	
	Compound 14	failed test for normality	Kruskal Wallis		7	0.4336	
	Compound 16	normal	One-way ANOVA		7	0.9003	
4B	WT		Two-way ANOVA	Dunnett	4	<0.0001	9: <0.0001, 10: 0.002, 13: <0.0001
	G544D		Two-way ANOVA	Dunnett	4	0.0001	9: <0.0001, 10: <0.0001, 13: <0.0001
	R406H		Two-way ANOVA	Dunnett	4	<0.0001	9: 0.0228, 10: 0.0167, 13: 0.0146
4C	Compound 9	failed test for normality	Kruskal Wallis	Dunn	9	0.0001	WT: 0.001, R406H: 0.0001, G544D: 0.0028
	Compound 10	normal	One-way ANOVA	Dunnett	9	0.0004	WT: 0.0072, R406H: 0.0002, G544D: 0.0021
	Compound 13	failed test for normality	Kruskal Wallis	Dunn	9	0.0001	WT: 0.0005, R406H: 0.0003, G544D: 0.0025
5B	Compound 9	normal	One-way ANOVA	Dunnett	DMSO: 53, 9-0.25uM: 11; 9-1uM, 9-5uM: 15; 9-20uM, 9-100uM: 14.	0.0016	0.25 µM: 0.9945, 1 µM: 0.0008, 5 µM: 0.9999, 20 µM: 0.9844; 100 µM: 0.8603
	Compound 10	failed test for normality	Kruskal Wallis	Dunn	DMSO: 53, 10-0.25uM, -1uM, -5uM: 11; 10-20uM, -100uM: 10	<0.0001	0.25 µM: >0.9999, 1 µM: >0.9999, 5 µM: >0.9999, 20 µM: 0.0052; 100 µM: 0.0216
	Compound 13	normal	One-way ANOVA	Dunnett	DMSO: 53, 13-0.25uM: 11; 13-1uM:16; 13-5uM, 13-20uM:14; 13-100uM: 15.	0.0095	0.25 µM: 0.9248, 1 µM: >0.9999, 5 µM: 0.0021, 20 µM: 0.0925; 100 µM: 0.6689
S4C	Compound 9	failed test for normality	Kruskal Wallis	Dunn	DMSO: 88; 9-0.25uM: 16; 9-1uM, 9-5uM, 9-20uM: 23; 9-100uM: 22.	0.0592	
	Compound 10	failed test for normality	Kruskal Wallis	Dunn	DMSO: 88; 10-0.25uM: 13; 10-1uM, 10-5uM: 21 10-20uM:20; 10-100uM: 16	<0.0001	0.25 µM: >0.9999, 1 µM: >0.9999, 5 µM: 0.5727, 20 µM: 0.0027; 100 µM: >0.0001
	Compound 13	normal	One-way ANOVA	Dunnett	DMSO: 88; 13-0.25uM: 9; 13-1uM: 25; 13-5uM:21; 13-20uM:20; 13-100uM: 21.	0.001	0.25 µM: 0.1787, 1 µM: 0.6498, 5 µM: 0.0323, 20 µM: 0.1403; 100 µM: 0.983
6D	G544D	normal	One-way ANOVA	Dunnett	cre only: 3, WT: 10, DMSO: 7, 9-1 µM: 8, 9-5 µM: 6, 10-1 µM: 7, 10-5 µM: 6, 13-1 µM: 5, 13-5 µM: 6, 13-20 µM: 7, PB: 3	<0.0001	9-1 µM: 0.0008, 9-5 µM: 0.004, 10-1 µM: 0.0122, 10-5 µM: 0.0272, 13-1 µM: 0.9963, 13-5 µM: 0.0381, 13-20 µM: 0.4939, WT: <0.0001, cre: 0.9994, 4-PBA: <0.0001
	R406H	normal	One-way ANOVA	Dunnett	DMSO: 4, 9-1 µM: 4, 10-1 µM: 7, 10-5uM: 4, 13-5 µM: 7, 13-20 µM: 7	<0.0001	9-1 µM: 0.0048, 10-1 µM: 0.0013, 10-5 µM: 0.0088, 13-5 µM: 0.0048, 13-20 µM: 0.0067, WT: <0.0001, cre: 0.9996
7B	G544D, compound 9		Two-way ANOVA	NA		0.0429	
7C	G544D, compound 10		Two-way ANOVA	NA		0.0412	
7D	G544D, compound 13		Two-way ANOVA	NA		0.0237	

7E	R406H, compound 9		Two-way ANOVA	NA		0.0153	
7F	R406H, compound 10		Two-way ANOVA	NA		0.0116	
7G	R406H, compound 13		Two-way ANOVA	NA		0.0224	
7H	G544D	normal	One-way ANOVA	Dunnett	WT : 10, DMSO: 23, 9-1 µM: 11, 9-5 µM: 16, 9-20 µM: 14, 10-0.25 µM: 7, 10-1 µM: 8, 13-1 µM: 15, 13-5 µM: 21, 13-20 µM: 15	<0.0001	WT: <0.0001, 9-1 µM: 0.0118, 9-5 µM: <0.0001, 9-20 µM: 0.0006, 10-0.25 µM: 0.6372, 10-1 µM: 0.0364, 13-1 µM: <0.0001, 13-5 µM: 0.0005, 13-20 µM: 0.0018
7I	R406H	normal	One-way ANOVA	Dunnett	WT : 10, DMSO: 14, 9-1 µM: 17, 9-5 µM: 9, 9-20 µM: 10, 10-0.25 µM : 8, 10-1 µM : 9, 13-1 µM: 13, 13-5 µM: 12, 13-20 µM: 13	<0.0001	WT: <0.0001, 9-1 µM: 0.0002, 9-5 µM: 0.0006, 9-20 µM: 0.0006, 10-0.25 µM: 0.2267, 10-1 µM: 0.9963, 13-1 µM: 0.0407, 13-5 µM: 0.003, 13-20 µM: <0.0001
8A/B	G544D + compound 9		Two-way ANOVA	Dunnett	4 for every treatment group, WT: 3	<0.0001	Compared to control - WT: 0.0002, 1 µM: 0.0024, 5 µM: 0.014, 20 µM: 0.0781, 100 µM: 0.1485; Compared to WT - Control: 0.0002, 1 µM: 0.0019, 5 µM: 0.0053, 20 µM: 0.0144, 100 µM: 0.0065
8C/D	G544D + compound 10		Two-way ANOVA	Dunnett	4 for every treatment group, WT: 4	<0.0001	Compared to control - WT: 0.0001, 1 µM: 0.779, 5 µM: 0.4638, 20 µM: 0.44, 100 µM: 0.4862; Compared to WT - Control: 0.0001, 1 µM: 0.0007, 5 µM: 0.0084, 20 µM: 0.0001, 100 µM: 0.0166
8E/F	G544D + compound 13		Two-way ANOVA	Dunnett	3 for every treatment group, WT: 4	<0.0001	Compared to control - WT: 0.0266, 1 µM: 0.0929, 5 µM: 0.0421, 20 µM: 0.0253, 100 µM: 0.0183; Compared to WT - Control: 0.0013, 1 µM: 0.0277, 5 µM: 0.0001, 20 µM: 0.4972, 100 µM: 0.1802
8G/H	R406H + compound 9		Two-way ANOVA	Dunnett	4 for every treatment group, WT: 3	<0.0001	Compared to control - WT: 0.1174, 1 µM: 0.1522, 5 µM: 0.1484, 20 µM: 0.1312, 100 µM: 0.1522; Compared to WT - Control: 0.1111, 1 µM: 0.4219, 5 µM: 0.7608, 20 µM: 0.7608, 100 µM: 0.4219
8I/J	R406H + compound 10		Two-way ANOVA	Dunnett	4 for every treatment group, WT: 4	<0.0001	Compared to control - WT: 0.4584, 1 µM: 0.4584, 5 µM: 0.4584, 20 µM: 0.4584, 100 µM: 0.4584
8K/L	R406H + compound 13		Two-way ANOVA	Dunnett	4 for every treatment group, WT: 4	<0.0001	Compared to control - WT: 0.0013, 1 µM: 0.0182, 5 µM: 0.1259, 20 µM: 0.1899, 100 µM: 0.0003; Compared to WT - Control: 0.0013, 1 µM: 0.046, 5 µM: 0.103, 20 µM: 0.0846, 100 µM: 0.4387
8P	Soluble Pool	normal	One-way ANOVA	Dunnett	WT: 4; GD: 9-1uM, 9-5uM, 9-100uM, 10-5uM, 10-100uM, 13-1uM, 13-5uM, 13-20uM, 13-100uM: 4; DMSO, 4-PB, 9-20uM, 10-1uM, 10-20uM: 3	<0.0001	4-PBA: 0.0086, 9-1 µM: 0.0072, 9-5 µM: >0.9999, 9-20 µM: >0.9999, 9-100 µM: >0.9999, 10-1 µM: 0.2032, 10-5 µM: 0.3347, 10-20 µM: 0.2515, 10-100 µM: 0.49, 13-1 µM: <0.0001. 13-5 µM: >0.0001, 13-20 µM: 0.9424, 13-100 µM: 0.9896, control: <0.0001
	Insoluble Pool	normal	One-way ANOVA	Dunnett	WT: 4; GD: 9-1uM, 9-5uM, 9-100uM, 10-5uM, 10-100uM, 13-1uM, 13-5uM, 13-20uM, 13-100uM: 4; DMSO, 4-PB, 9-20uM, 10-1uM, 10-20uM: 3	<0.0001	all <0.0001
S6A	WT + compound 9		Two-way ANOVA	Dunnett	4 for every treatment group, WT: 3	0.8805	
S6B	WT + compound 10		Two-way ANOVA	Dunnett	4 for every treatment group, WT: 4	0.9773	
S6C	WT + compound 13		Two-way ANOVA	Dunnett	4 for every treatment group, WT: 4	0.9871	

Appendix Table S2. Binding site and docking scores for top compounds identified *in silico* that were tested for pharmacological activity.

#	Compound	Binding Site	Docking score
1	Levetiracetam (((S)-2-(2-Oxo-1-pyrrolidinyl)butyramide))	N/A (known anti-epileptic)	
2	5-Aminoimidazole-4-carboxamide 1-beta-D-Ribofuranoside	3	-7.499
3	Neamine	2	-7.181
4	(1R,3S)-3-(2-aminoethoxy)-7-(2,1,3-benzothiadiazol-5-ylmethyl)-7-azaspiro[3.5]nonan-1-ol	1	-7.922
5	5-(indol-1-ylmethyl)-N-(3-pyridylmethyl)-1H-pyrazole-3-carboxamide	1	-7.799
6	N-[(2-ethoxy-3-pyridyl)methyl]-5-(p-tolyl)-4H-pyrazole-3-carboxamide	1	-7.531
7	4-fluoro-N-[3-oxo-3-[[(1R)-1-(5-oxo-1,2,4-triazol-3-yl)ethyl]amino]propyl]benzamide	1	-7.428
8	2-[(4S)-1,3-dimethyl-2,5-dioxo-imidazolidin-4-yl]-N-[(3-phenyl-1H-1,2,4-triazol-5-yl)methyl]acetamid	1	-8.131
9	2-[[(1R,3S)-1-hydroxy-3-methoxy-7-azaspiro[3.5]nonan-7-yl]methyl]-3,5-dimethyl-1H-pyridin-4-one	1	-7.731
10	(2S)-1-[4-[(benzothiophen-3-ylmethylamino)methyl]-1-piperidyl]propan-2-ol	2	-7.828
11	2-[(3S)-3-[[(4-methyl-2-pyridyl)amino]methyl]pyrrolidin-1-yl]-N-(2-thienylmethyl)acetamide	2	-7.643
12	4-[(E)-[(3S)-3-(trifluoromethyl)-3H-1,2,4-triazol-5-yl]azo]phenol	2	-7.268
13	3-[2-[[1-[(2S)-2-hydroxypropyl]-4-piperidyl]methylamino]ethyl]imidazolidine-2,4-dione	2	-7.256
14	(2S)-3-methyl-N-[2-[(4-methyl-2-pyridyl)amino]ethyl]-3,8-diazaspiro[4.5]decane-2-carboxamide	2	-7.699
15	5-[1-[(2-butyl-1H-imidazol-4-yl)methyl]-4-piperidyl]-4-ethyl-2-methyl-1,2,4-triazol-3-one	2	-7.583
16	4-[2-[[(3R)-3-hydroxypyrrolidin-3-yl]methylamino]ethyl]benzenesulfonamide	3	-8.924
17	2-[[(4S)-2-(3-methoxyanilino)-4H-purin-6-yl]amino]ethanol	3	-8.874
18	N-[(2,3-dimethoxyphenyl)methyl]-6-[(3S)-3-piperidyl]pyrimidin-4-amine	3	-8.001
