# Supplementary material for: Targeted stabilization of Munc18‐1 function via pharmacological chaperones
Source: EMBO Mol Med. 2020 Dec 17;13(1):e12354. doi: 10.15252/emmm.202012354 (PMC7799358; doi:10.15252/emmm.202012354)
Supplement: Supplementary file 2 — Source Data for Appendix [file EMMM-13-e12354-s010.zip › EMM-2020-12354-V3-Figure_S2_Source_Data-sd.pdf]

Annotated

S2A

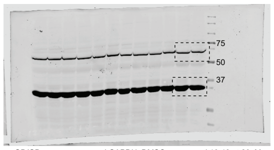

GD/GD neurons, myc and GAPDH: DMSO, compound 10-18 at 20uM and H_2O and 4-PB

S2B

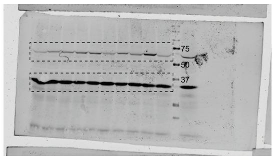

Heterozygous neurons, munc18-1 and GAPDH: compound 1-9 at 20uM, DMSO

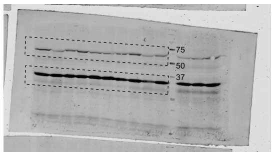

Heterozygous neurons, munc18-1 and GAPDH: compound 10-18 at 20uM, DMSO

S2C

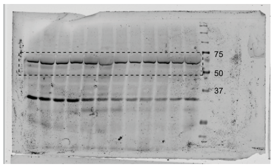

Heterozygous neurons, munc18-1: DMSO, compound 9 at 0.25, 1, 5, 20, 100uM and compound 10 at 0.25, 1, 5, 20, 100uM

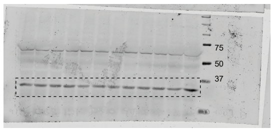

Heterozygous neurons, GAPDH: DMSO, compound 9 at 0.25, 1, 5, 20, 100uM and compound 10 at 0.25, 1, 5, 20, 100uM

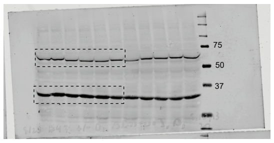

Heterozygous neurons, munc18-1 and GAPDH: DMSO, compound 13 at 0.25, 1, 5, 20, 100uM

S2D

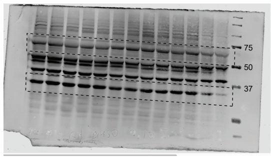

RH/RH neurons, myc, GAPDH: DMSO, compound 9 at 0.25, 1, 5, 20, 100, 250 uM and compound 10 at 0.25, 1, 5, 20, 100, 250uM

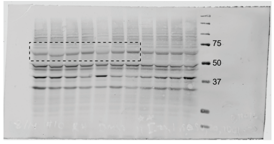

RH/RH neurons, myc: DMSO, compound 11 at 0.25, 1, 5, 20, 100, 250 uM

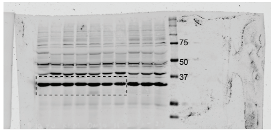

RH/RH neurons, GAPDH: DMSO, compound 11 at 0.25, 1, 5, 20, 100, 250 uM

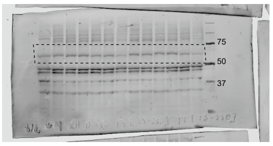

RH/RH neurons, myc: DMSO, compound 13 at 0.25, 1, 5, 20, 100, 250 uM and compound 14 at 0.25, 1, 5, 20, 100, 250uM

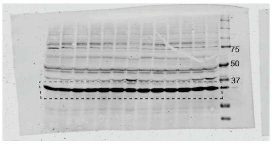

RH/RH neurons, GAPDH: DMSO, compound 13 at 0.25, 1, 5, 20, 100, 250 uM and compound 14 at 0.25, 1, 5, 20, 100, 250uM

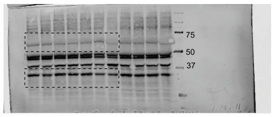

RH/RH neurons, myc GAPDH: DMSO, compound 16 at 0.25, 1, 5, 20, 100, 250 uM

Not Annotated

S2A

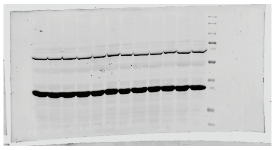

GD/GD neurons, myc and GAPDH: DMSO, compound 10-18 at 20uM and H_2O and 4-PB

S2B

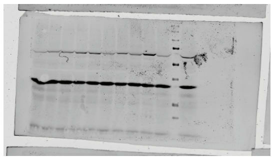

Heterozygous neurons, munc18-1 and GAPDH: compound 1-9 at 20uM, DMSO

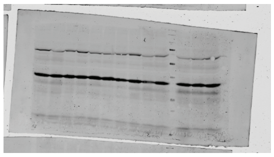

Heterozygous neurons, munc18-1 and GAPDH: compound 10-18 at 20uM, DMSO

S2C

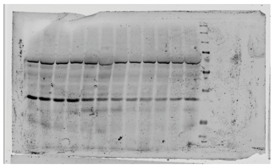

Heterozygous neurons, munc18-1: DMSO, compound 9 at 0.25, 1, 5, 20, 100uM and compound 10 at 0.25, 1, 5, 20, 100uM

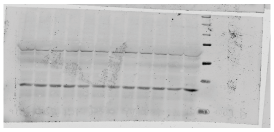

Heterozygous neurons, GAPDH: DMSO, compound 9 at 0.25, 1, 5, 20, 100uM and compound 10 at 0.25, 1, 5, 20, 100uM

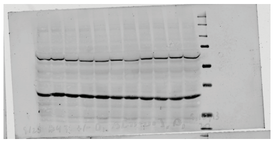

Heterozygous neurons, munc18-1 and GAPDH: DMSO, compound 13 at 0.25, 1, 5, 20, 100uM

S2D

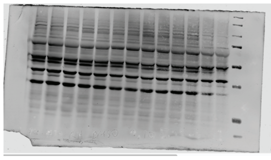

RH/RH neurons, myc, GAPDH: DMSO, compound 9 at 0.25, 1, 5, 20, 100, 250 uM and compound 10 at 0.25, 1, 5, 20, 100, 250uM

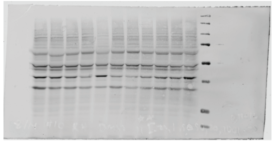

RH/RH neurons, myc: DMSO, compound 11 at 0.25, 1, 5, 20, 100, 250 uM

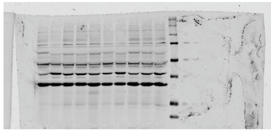

RH/RH neurons, GAPDH: DMSO, compound 11 at 0.25, 1, 5, 20, 100, 250 uM

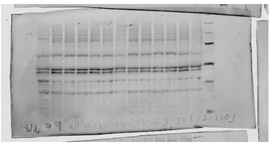

RH/RH neurons, myc: DMSO, compound 13 at 0.25, 1, 5, 20, 100, 250 uM and compound 14 at 0.25, 1, 5, 20, 100, 250uM

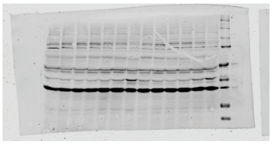

RH/RH neurons, GAPDH: DMSO, compound 13 at 0.25, 1, 5, 20, 100, 250 uM and compound 14 at 0.25, 1, 5, 20, 100, 250uM

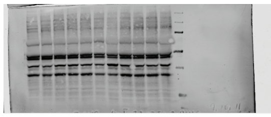

RH/RH neurons, myc, GAPDH: DMSO, compound 16 at 0.25, 1, 5, 20, 100, 250 uM
