# Supplementary figures and images for: Targeted stabilization of Munc18‐1 function via pharmacological chaperones
Source: EMBO Mol Med. 2020 Dec 17;13(1):e12354. doi: 10.15252/emmm.202012354 (PMC7799358; doi:10.15252/emmm.202012354)

**Figure 1C**

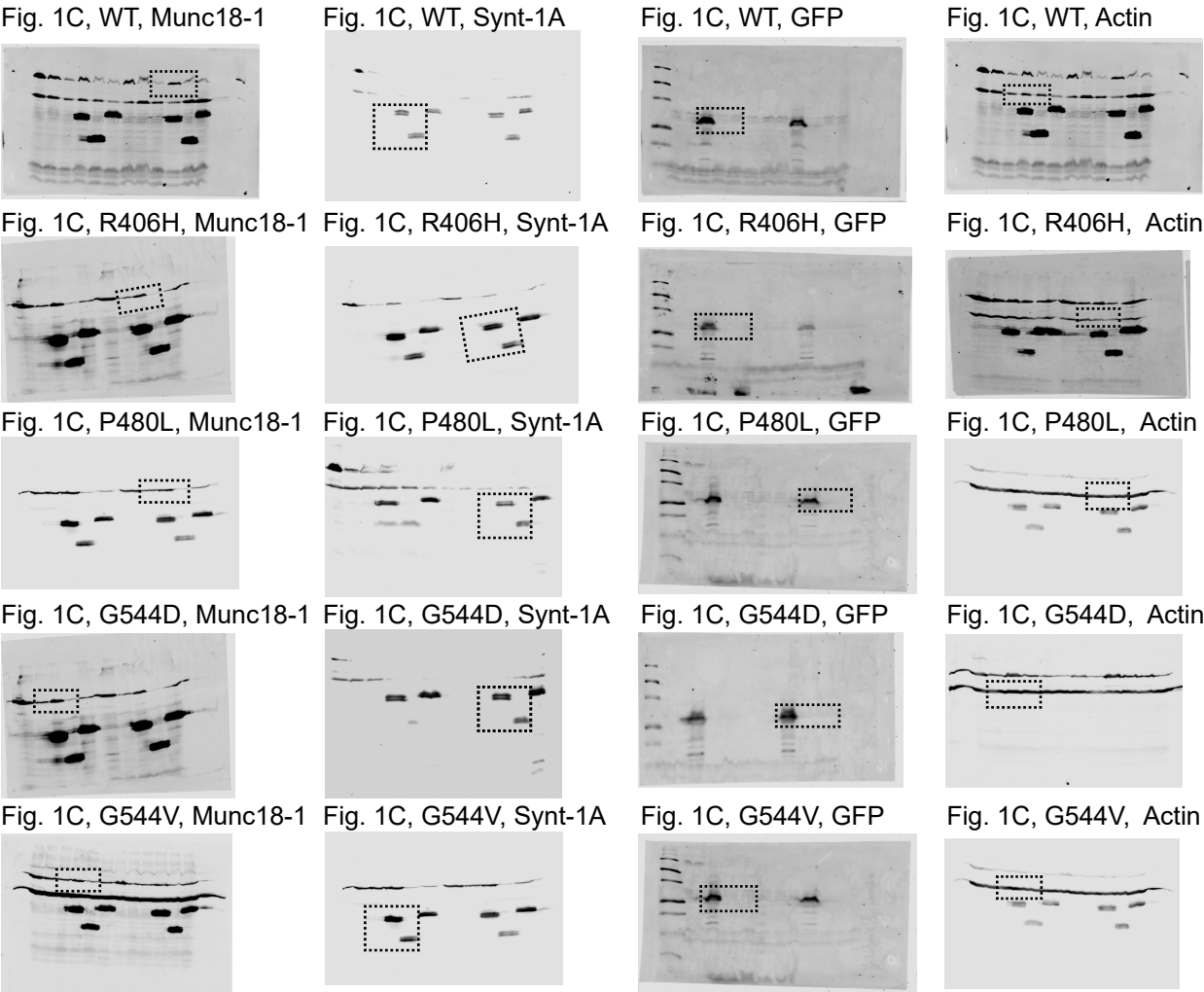

**Figure 1D**

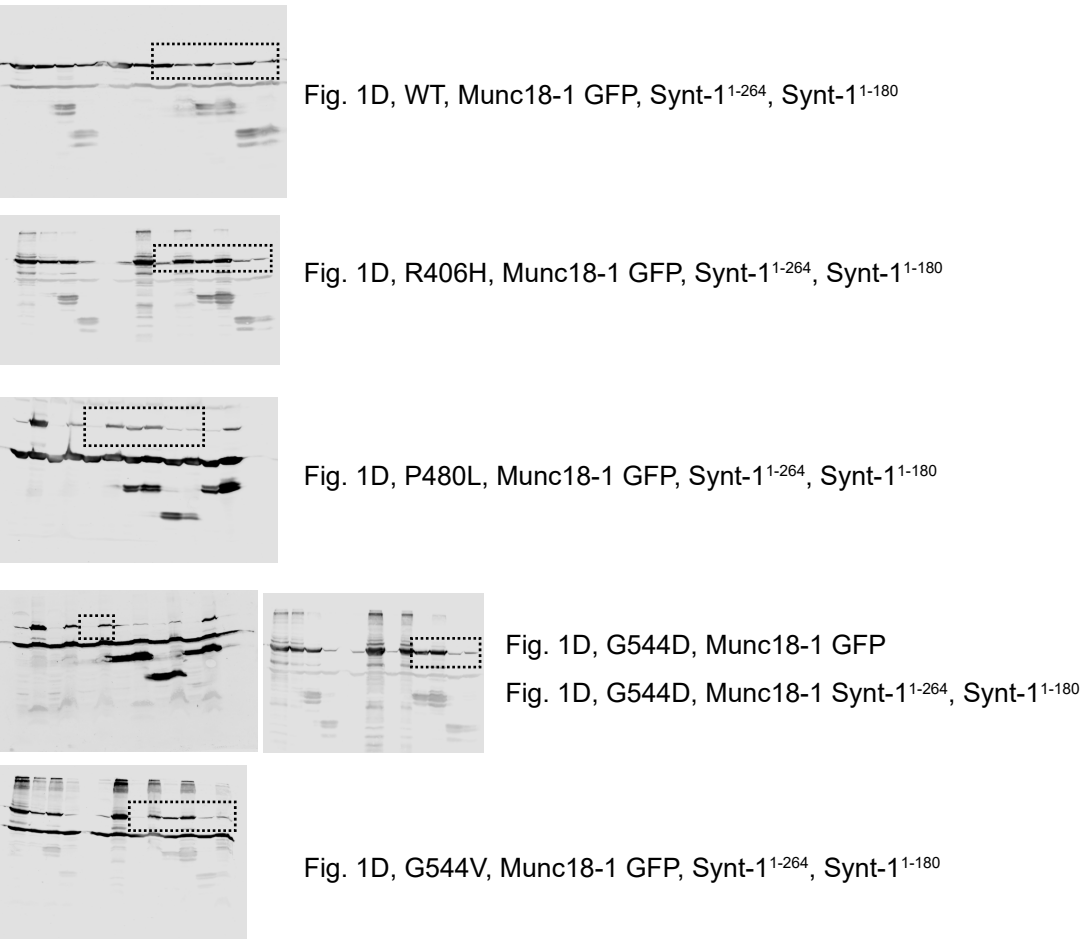

Supplement: Supplementary file 4 — Source Data for Figure 1 [file EMMM-13-e12354-s002.zip › EMM-2020-12354-V3-Figure_1_Source_Data-sd(1).pdf]

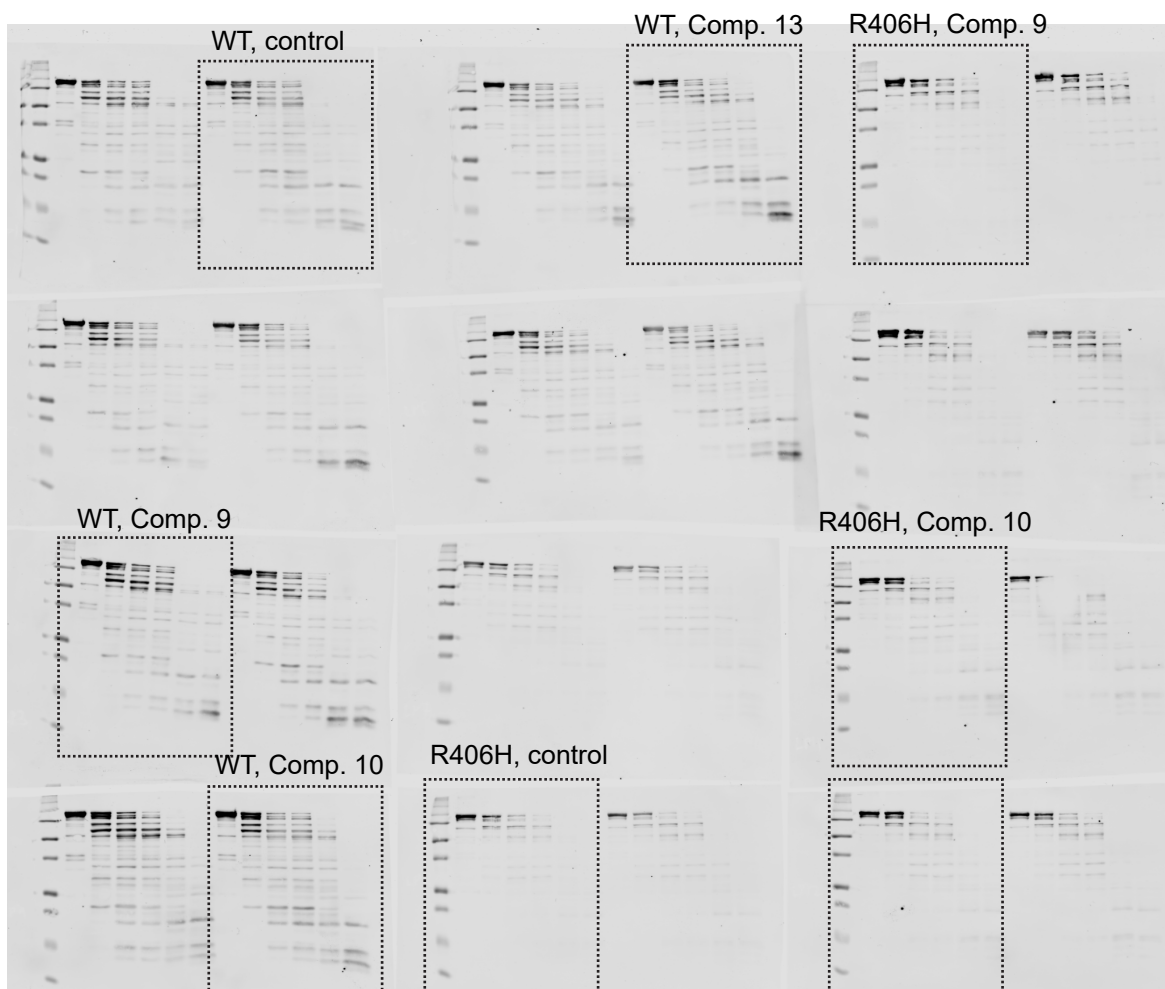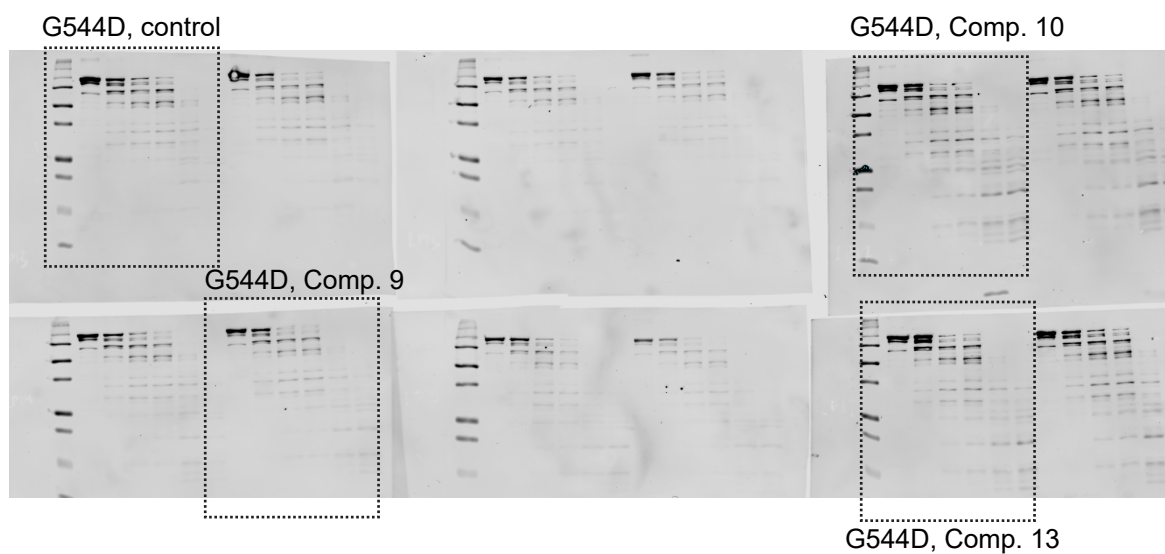

Supplement: Supplementary file 6 — Source Data for Figure 4 [file EMMM-13-e12354-s004.zip › EMM-2020-12354-V3-Figure_4_Source_Data-sd.pdf]
