# Supplementary material for: Targeted stabilization of Munc18‐1 function via pharmacological chaperones
Source: EMBO Mol Med. 2020 Dec 17;13(1):e12354. doi: 10.15252/emmm.202012354 (PMC7799358; doi:10.15252/emmm.202012354)
Supplement: Supplementary file 5 — Source Data for Figure 3 [file EMMM-13-e12354-s003.zip › EMM-2020-12354-V3-Figure_3_Source_Data-sd.pdf]

Annotated

A

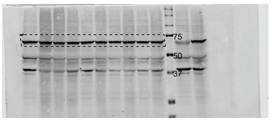

GD/GD neurons, myc: compound 1-9 at 20uM, DMSO

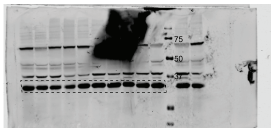

GD/GD neurons, GAPDH: compound 1-9 at 20uM, DMSO

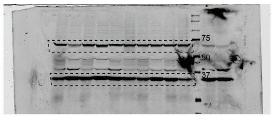

GD/GD neurons, myc and GAPDH: compound 10-18 at 20uM, DMSO

C

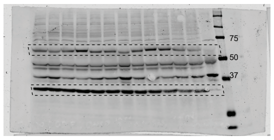

GD/GD neurons, myc, GAPDH: compound 9 at 0.25, 1, 5, 20, 100, 250 uM
and compound 10 at 0.25, 1, 5, 20, 100, 250uM

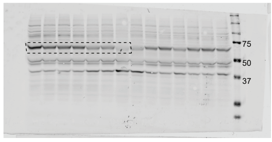

GD/GD neurons, myc : compound 11 at 0.25, 1, 5, 20, 100, 250 uM

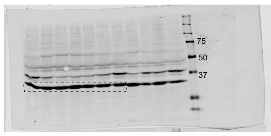

GD/GD neurons, GAPDH : compound 11 at 0.25, 1, 5, 20, 100, 250 uM

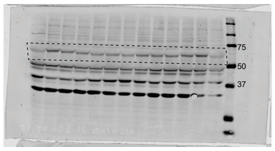

GD/GD neurons, myc : compound 13 at 0.25, 1, 5, 20, 100, 250 uM
and compound 14 at 0.25, 1, 5, 20, 100, 250uM

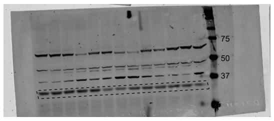

GD/GD neurons, GAPDH: compound 13 at 0.25, 1, 5, 20, 100, 250 uM
and compound 14 at 0.25, 1, 5, 20, 100, 250uM

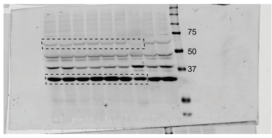

GD/GD neurons, myc, GAPDH: compound 16 at 0.25, 1, 5, 20, 100, 250 uM

Not Annotated

A

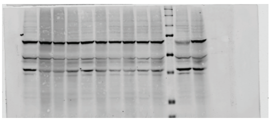

GD/GD neurons, myc: compound 1-9 at 20uM, DMSO

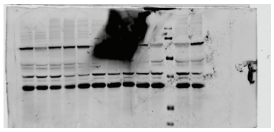

GD/GD neurons, GAPDH: compound 1-9 at 20uM, DMSO

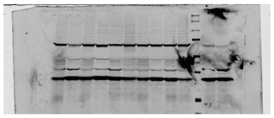

GD/GD neurons, myc and GAPDH: compound 10-18 at 20uM, DMSO

C

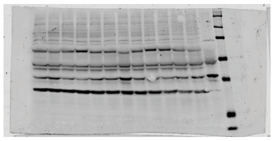

GD/GD neurons, myc, GAPDH: compound 9 at 0.25, 1, 5, 20, 100, 250 uM
and compound 10 at 0.25, 1, 5, 20, 100, 250uM

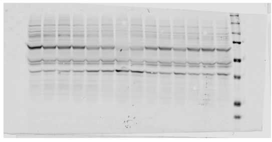

GD/GD neurons, myc : compound 11 at 0.25, 1, 5, 20, 100, 250 uM

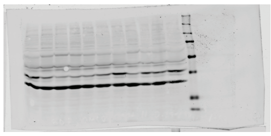

GD/GD neurons, GAPDH : compound 11 at 0.25, 1, 5, 20, 100, 250 uM

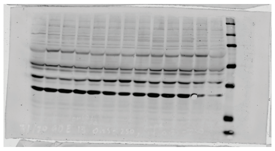

GD/GD neurons, myc : compound 13 at 0.25, 1, 5, 20, 100, 250 uM
and compound 14 at 0.25, 1, 5, 20, 100, 250uM

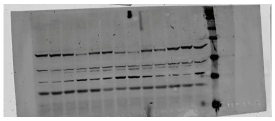

GD/GD neurons, GAPDH: compound 13 at 0.25, 1, 5, 20, 100, 250 uM
and compound 14 at 0.25, 1, 5, 20, 100, 250uM

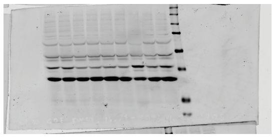

GD/GD neurons, myc, GAPDH: compound 16 at 0.25, 1, 5, 20, 100, 250 uM
