# Supplementary material for: Targeted stabilization of Munc18‐1 function via pharmacological chaperones
Source: EMBO Mol Med. 2020 Dec 17;13(1):e12354. doi: 10.15252/emmm.202012354 (PMC7799358; doi:10.15252/emmm.202012354)
Supplement: Supplementary file 8 — Source Data for Figure 6 [file EMMM-13-e12354-s006.zip › EMM-2020-12354-V3-Figure_6_Source_Data-sd.pdf]

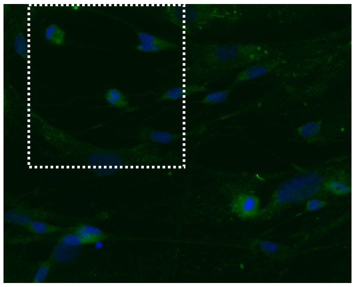
Fig. 6A, cre (turned 180°)

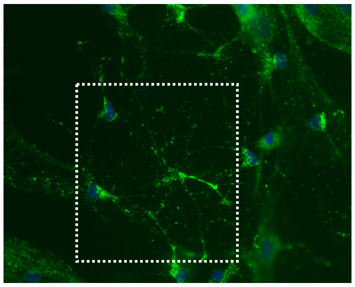
Fig. 6A, WT (turned -90°)

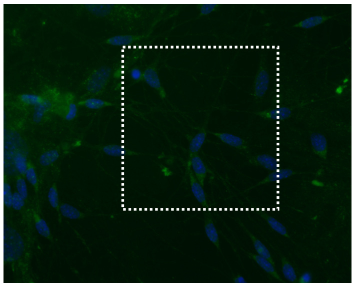
Fig. 6B, GD + DMSO

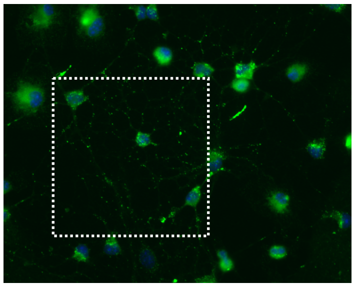
Fig. 6B, GD + 9-1 μM (turned -90°)

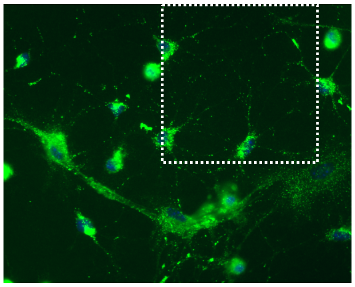
Fig. 6B, GD + 9-5 μM

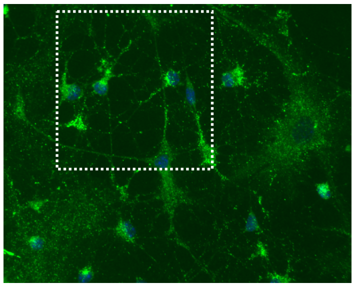
Fig. 6B, GD + 10-1 μM

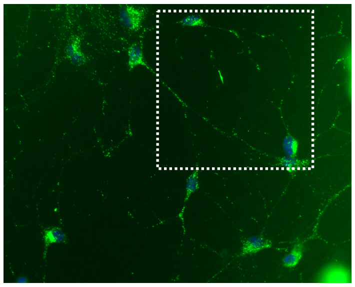
Fig. 6B, GD + 10-5 μM (turned -90°)

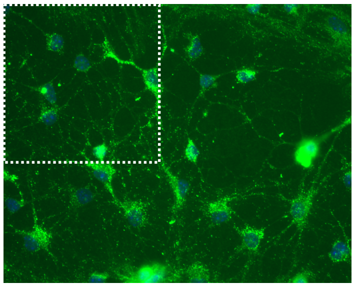
Fig. 6B, GD + 4-PBA (turned 90°)

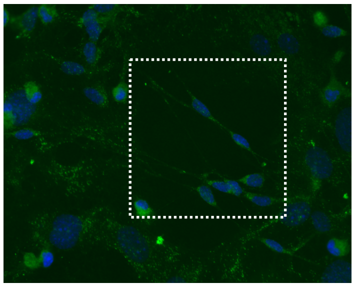
Fig. 6B, GD + 13-1 μM

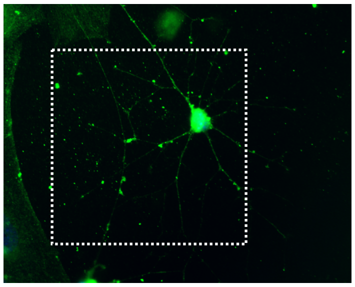
Fig. 6B, G544D + 13-5 μM

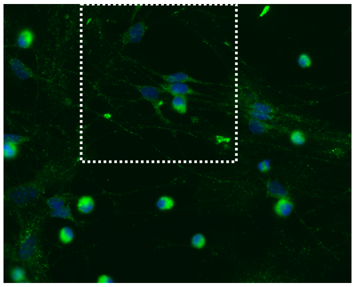
Fig. 6B, GD + 13-20 μM (turned 90°)

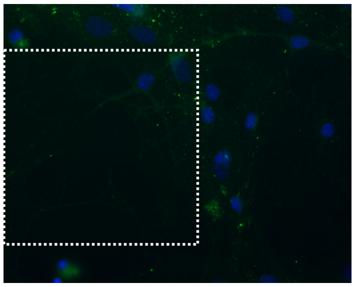
Fig. 6C, R406H + DMSO

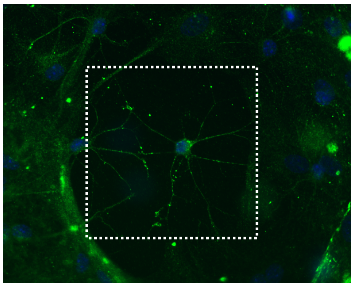
Fig. 6C, R406H + 9-1 μM

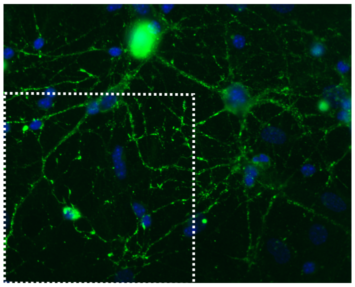
Fig. 6C, R406H + 10-1 μM

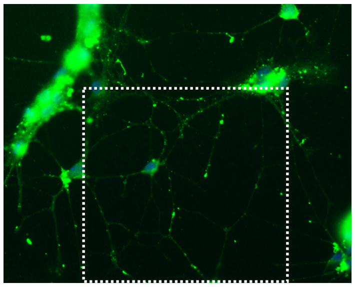
Fig. 6C, R406H + 10-5 μM

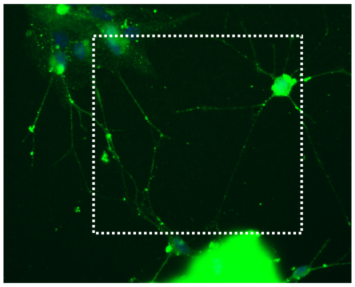
Fig. 6C, R406H + 13-5 μM

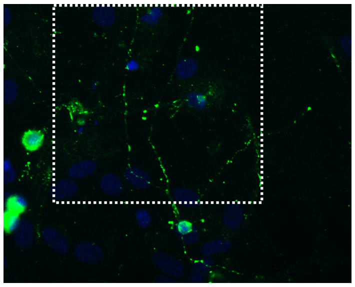
Fig. 6C, R406H + 13-20 μM
