# Supplementary material for: Targeted stabilization of Munc18‐1 function via pharmacological chaperones
Source: EMBO Mol Med. 2020 Dec 17;13(1):e12354. doi: 10.15252/emmm.202012354 (PMC7799358; doi:10.15252/emmm.202012354)
Supplement: Supplementary file 9 — Source Data for Figure 7 [file EMM-2020-12354-V3-Figure_7_Source_Data-sd.pdf › EMM-2020-12354-V3-Figure_7_Source_Data-sd.pdf]

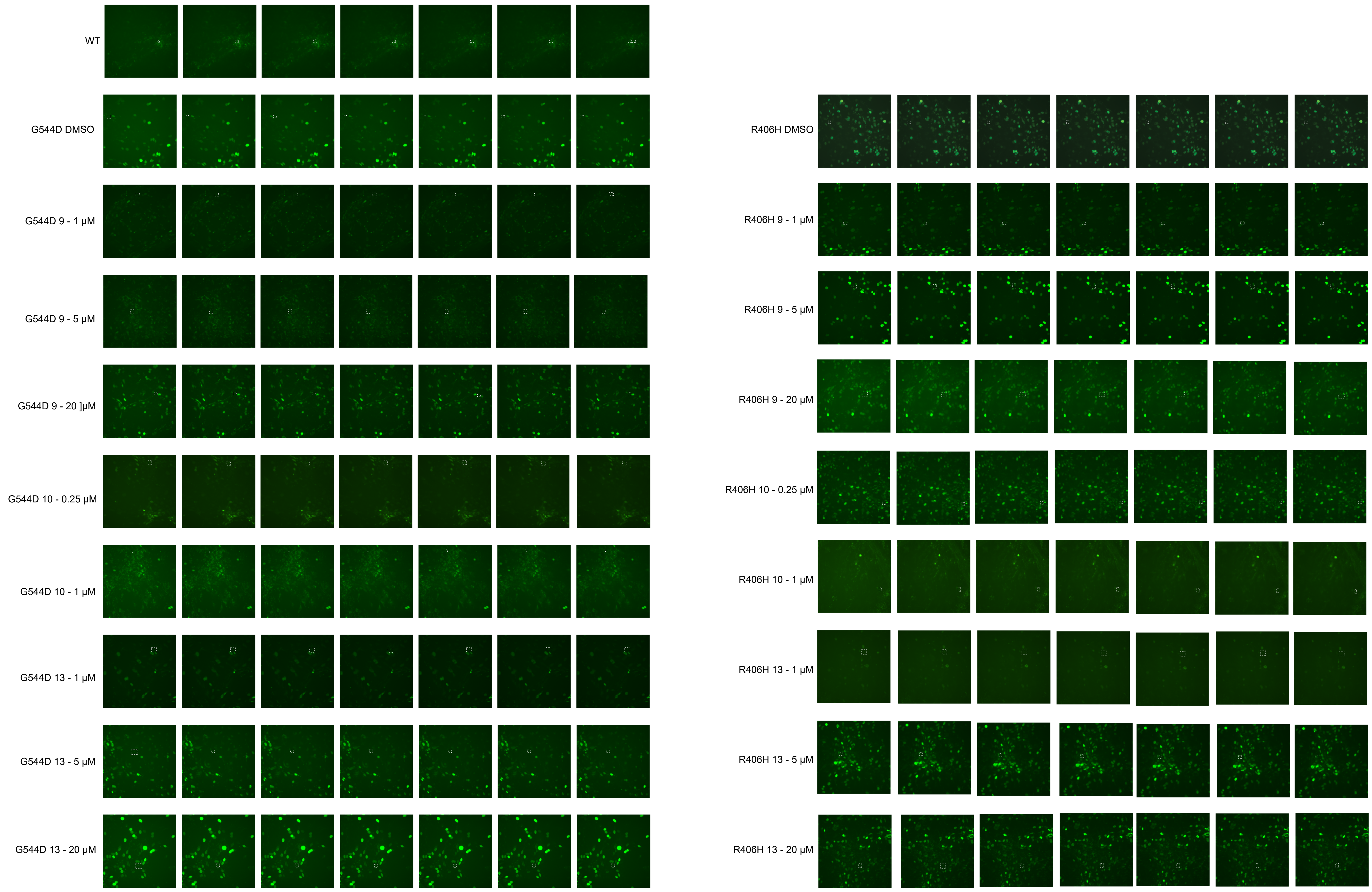
WT
G544D DMSO
G544D 9 - 1 μM
G544D 9 - 5 μM
G544D 9 - 20]μM
G544D 10 - 0.25 μM
G544D 10 - 1 μM
G544D 13 - 1 μM
G544D 13 - 5 μM
G544D 13 - 20 μM
R406H DMSO
R406H 9 - 1 μM
R406H 9 - 5 μM
R406H 9 - 20 μM
R406H 10 - 0.25 μM
R406H 10 - 1 μM
R406H 13 - 1 μM
R406H 13 - 5 μM
R406H 13 - 20 μM
